# C-Reactive Protein Correlates with Negative Symptoms in Patients with Schizophrenia

**DOI:** 10.3389/fpubh.2017.00360

**Published:** 2018-01-22

**Authors:** Ted Boozalis, Antonio L. Teixeira, Raymond Young-Jin Cho, Olaoluwa Okusaga

**Affiliations:** ^1^Department of Psychiatry and Behavioral Sciences, University of Texas Health Science Center at Houston, Houston, TX, United States; ^2^University of Texas Harris County Psychiatric Center, Houston, TX, United States; ^3^Department of Psychiatry and Behavioral Sciences, Baylor College of Medicine, Houston, TX, United States

**Keywords:** schizophrenia, C-reactive protein, inflammation, psychosis, PANSS, cognition, NIH toolbox

## Abstract

Peripheral and CNS-localized inflammatory processes are hypothesized to contribute to the complex pathophysiology of schizophrenia. Elevated levels of the acute phase reactant C-reactive protein (CRP) have been observed in schizophrenia, yet relatively few studies have investigated the association between this inflammatory biomarker and psychotic symptoms in schizophrenia. This study is a pilot cross-sectional analysis investigating the relation of plasma CRP levels and the positive and negative symptoms of schizophrenia (the primary aim), assessed by the Positive and Negative Syndrome Scale (PANSS). A secondary analysis was also performed evaluating the potential association of CRP with cognitive function using the NIH Toolbox Cognitive Test Battery. After adjusting for age, sex, race, and body mass index, a positive correlation was observed between CRP and PANSS negative symptoms (rho = 0.37, *p* = 0.05). There was no correlation between plasma CRP and any of the NIH Toolbox measures of cognitive function in the unadjusted or adjusted analyses. Though limited by a relatively small sample size and the unavailability of longitudinal data, the correlation between CRP and psychopathology in this sample of patients supports a role for inflammation in the pathophysiology of schizophrenia.

## Introduction

Peripheral and CNS-localized markers of immunopathology, including cytokine expression and microglial activation, have been associated with schizophrenia (1–5), a psychotic disorder of complex genetic and environmental pathogenesis. Prior studies have indicated that systemic inflammatory mechanisms can influence neural circuits (6–10), and an evolving genetic-vascular-inflammatory theory of schizophrenia suggests that genetically regulated inflammatory reactions cause progressive microvascular damage to the central nervous system in response to environmental stressors (11). Furthermore, animal models have shown that chronically elevated brain levels of pro-inflammatory cytokines may lead to abnormal neural connectivity of the developing brain which could contribute to the emergence of psychosis. Subsequently, and in an attempt to target the low-grade neuroinflammation hypothesized to be present in the illness, adjunctive treatment of schizophrenia with anti-inflammatory agents has been evaluated in a number of studies albeit with mixed results (12–14). However, the relationship between inflammatory processes and schizophrenia remains incompletely understood.

The acute phase reactant C-reactive protein (CRP) is a non-specific serum marker of inflammation. Elevated blood levels of CRP have been observed in schizophrenia (15–17), and elevated CRP in adolescence has been associated with subsequent development of schizophrenia in adulthood (18). A causal association between chronic systemic inflammation and schizophrenia has been suggested (19) but is yet to be confirmed. Moreover, inflammation during prenatal and perinatal neurodevelopment, as measured by elevated levels of IL-8 and TNF-alpha in maternal plasma, has been implicated as a risk factor for subsequent development of schizophrenia in offspring (1, 20, 21). Furthermore, a negative correlation has been observed between several systemic inflammatory markers, including CRP, and general cognitive functioning (22, 23) as well as with all facets of working memory (22, 24) in patients with schizophrenia. Decreased levels of IL-10, an anti-inflammatory cytokine, have also been associated with cognitive impairment in first-episode medication-naïve patients with schizophrenia (25).

Based on the hypothesized link between inflammation and schizophrenia, a few studies have investigated the association between plasma CRP and psychotic symptoms in schizophrenia. Three studies found that patients with schizophrenia and higher levels of serum CRP scored higher on psychotic symptom severity rating scales in comparison to those patients with lower serum CRP levels (26–28). In a study by Akanji and colleagues, higher serum CRP levels were found in patients with catatonic features but, paradoxically, also in patients whose symptoms were in remission (29). In contrast, three separate studies did not find a significant association between CRP levels and positive or negative symptoms in patients with schizophrenia (24, 30, 31). A possible reason for these equivocal results could be the failure to statistically adjust for potential confounders. For example, age and body mass index (BMI) have been shown to be positively correlated with CRP levels (32–36), and it is therefore recommended that these two variables are adjusted for in any analysis evaluating the relationship between CRP levels and psychotic symptoms.

To add to the relatively sparse literature evaluating the association between CRP and psychotic symptoms, we have carried out a cross-sectional analysis of plasma CRP levels and positive and negative symptoms in a pilot sample of 39 patients with schizophrenia. To overcome the primary potential confounds of prior studies, we statistically adjusted for important confounders including age, sex, race, and BMI. We hypothesized that plasma CRP would positively correlate with the positive, negative, general psychopathology, and total psychotic symptoms of schizophrenia. We also carried out a secondary exploratory analysis evaluating the association of CRP with cognitive function as assessed by the NIH Toolbox Cognitive Test Battery (37).

## Materials and Methods

### Patient Sample

The institutional review board of The University of Texas Health Science Center, Houston, Texas approved this study. This research was completed in conformity with the latest version of the Declaration of Helsinki. All study participants completed a written informed consent form after the study was described to them.

Inpatients with a diagnosis of schizophrenia were recruited from a hospital associated with a university department of psychiatry. All patients had a prior history of multiple episodes of psychosis, and all were being treated with antipsychotic medications at the time of data collection for this study. The following demographic information was obtained: age, sex, race BMI, and level of education. The following inclusion criteria were employed: age ranging from 18 to 60 years, prior documented diagnosis of schizophrenia by DSM-5 criteria, and a negative urine pregnancy test for female patients. The diagnosis of schizophrenia was subsequently confirmed with the Mini-International Neuropsychiatric interview, version 5 (38). The following criteria were employed for exclusion from this study: current endorsement of suicidal or homicidal ideations, prior diagnosis of other cognitive disorders, any current infectious diagnosis, any diagnosis of a primary inflammatory condition, any current use of corticosteroids or non-steroidal anti-inflammatory medications, any recent or current use of warfarin or anticoagulant medications, and a urine drug screen positive for psychostimulant drugs.

### Sample Collection and CRP Measurement

Fasting venous blood was collected from patients. Plasma CRP levels were then measured *via* enzyme-linked immunosorbent assays (Sigma-Aldrich, MO, USA) in conformance with manufacturer instructions. Samples were diluted 1:20,000. Optical density values for the samples were then compared to standard curves ranging from 0 to 600 pg/ml. The minimum detectable dose of human CRP in this assay is 2 pg/ml.

### Psychotic Symptom and Cognitive Assessment in Patients

The Positive and Negative Syndrome Scale (PANSS) (39) was used for the psychopathological assessment of the patients, recording the positive syndrome score, negative syndrome score, general psychopathology score, and total PANSS score. Additionally, the NIH Toolbox Cognitive Test Battery (37) was used to assess the following aspects of cognitive function: working memory (*via* the List Sorting Working Memory Test), episodic memory (*via* the Picture Sequence Memory Test), processing speed (*via* the Pattern Comparison Process Speed Test), executive function (*via* the Dimensional Change Card Sort Test as well as the Flanker Inhibitory Control and Attention Test), and language ability (*via* the Oral Reading Recognition Test and Picture Vocabulary Test).

### Statistical Analysis

Descriptive statistics are presented as mean (with standard deviation) and median as appropriate. The distribution of CRP was skewed and thus for the primary aim (the association between CRP and psychotic symptom severity), Spearman rank correlations were calculated; similar analyses were carried out for the secondary aim (the association of CRP with cognitive measures). Based on prior studies showing positive correlations between systemic inflammation (and hence plasma CRP) and aging (32, 33) as well as BMI (34–36), we calculated nonparametric partial correlations (40) adjusted for age, sex, race, and BMI. By definition, partial correlations are estimates of the strength and direction of the linear relationship (i.e., correlation) between two variables (usually continuous variables) while making adjustment for the effect of one or more other variables. We calculated the nonparametric correlations adjusted for the aforementioned confounders (i.e., nonparametric partial correlations) by running a script (code) in SPSS syntax. For the primary analysis (i.e., primary aim), significance was set at alpha = 0.05. However, to adjust for multiple comparisons in the secondary analysis (eight different cognitive measures), the significance was set at 0.01. All tests were two-tailed. Data analysis was performed using IBM SPSS version 20 (IBM Corp., Armonk, NY, USA).

## Results

### Demographic and Clinical Characteristics of the Sample

Table 1 presents the demographic and clinical characteristics of the patient sample (*n* = 39) enrolled in this study. The sample population included more males (74.4%) than females (25.6%). Mean age was 32.7 years old (SD = 12.11), and mean BMI was 27.33 (SD = 5.22). All patients included in this study had a diagnosis of schizophrenia, as well as a history of multiple episodes of psychosis. All were being treated with antipsychotic medications at the time of data collection.

**Table 1:** Demographic and clinical characteristics of the sample of 39 patients with schizophrenia.

Demographic and clinical variables	Patient sample
Mean age ± SD	32.79 ± 12.05
**Gender**	
Male, *n* (%)	29 (74.4%)
Female, *n* (%)	10 (25.6%)
**Race**	
White, *n* (%)	11 (28.2%)
Black, *n* (%)	19 (48.7%)
Hispanic, *n* (%)	8 (20.5%)
Asian, *n* (%)	1 (2.6%)
**Education level**	
No high-school graduation, *n* (%)	7 (20.0%)
Graduated high-school (or equivalent), *n* (%)	10 (28.6%)
Part college, *n* (%)	14 (40.0%)
Graduated > 2 years of college, *n* (%)	4 (11.4%)
Mean body mass index ± SD	27.27 ± 5.19
Median C-reactive protein (ng/ml)	0.19

### CRP and Psychotic Symptoms

In the unadjusted analyses, a positive correlation was observed between CRP and the PANSS negative symptom subscale (rho = 0.41, *p* = 0.012) and between CRP and PANSS general psychopathology subscale (Table 2; rho = 0.36, *p* = 0.029). While a trend toward significance was observed in the correlation between CRP and total PANSS score (rho = 0.32, *p* = 0.059), there was no correlation between CRP and positive symptoms (rho = −0.21, *p* = 0.223). Following adjustment for potential confounders (age, sex, race, and BMI), CRP maintained a significant correlation with the PANSS negative symptom subscale (Table 2; rho = 0.37, *p* = 0.050), but did not correlate with any of the other PANSS subscales or with the total PANSS score (Table 2).

**Table 2:** Spearman correlation of C-reactive protein (CRP) and psychotic symptom scores, before and after adjustment for age, sex, race, and body mass index.

	Spearman’s rho (unadjusted)	*p*-value	Spearman’s rho (adjusted)	*p*-value
	
	CRP		CRP	
Positive and Negative Syndrome Scale (PANSS) positive symptoms	0.21	0.223	−0.26	0.186
PANSS negative symptoms	0.41	0.012	**0.37^a^**	0.050
PANSS General Psychopathology Score	0.36	0.029	0.27	0.161
Total PANSS Score	0.32	0.059	0.21	0.302

*^a^Statistically significant correlation after adjusting for potential confounders*.

### CRP and Cognitive Function

There was no correlation observed between plasma CRP and any of the NIH Toolbox measures of cognitive function in unadjusted and adjusted analyses (Table 3).

**Table 3:** Spearman correlation of C-reactive protein (CRP) and scores on the NIH Toolbox Cognitive Test Battery before and after adjustment for age, gender, race, and body mass index.

	Spearman’s rho (unadjusted)	*p*-value	Spearman’s rho (adjusted)	*p*-value

	CRP		CRP	
Cognition fluid composite	−0.03	0.900	0.20	0.396
Picture vocabulary	−0.30	0.103	−0.10	0.647
Flanker inhibitory control and attention	0.14	0.477	0.27	0.226
Sorting working memory	0.02	0.937	0.09	0.689
Dimensional change card sort	0.01	0.977	0.18	0.405
Pattern comparison process speed	0.00	0.996	0.05	0.835
Picture sequence memory	−0.24	0.214	−0.06	0.790
Oral reading recognition	−0.18	0.343	−0.08	0.720

## Discussion

This study found a positive correlation between plasma CRP and the negative symptoms of schizophrenia. We also found a positive correlation between CRP and the PANSS general psychopathology subscale in the unadjusted analysis but this finding was not robust as it did not persist after adjusting for potential confounders.

Based on the research carried out so far on the potential associations between CRP and specific psychotic domains of schizophrenia, no definite conclusions can be drawn and further exploration of this topic is needed. Some studies of CRP and symptom severity in schizophrenia have suggested that CRP levels rise during acute psychotic episodes (16) and that higher CRP levels are associated with heightened severity of psychosis (26–28) and with exacerbation of negative symptoms and general psychopathology (28). In one study, CRP was higher in patients with prominent catatonic features, although the same study found that CRP was highest during remission of symptoms (29). Others have found that CRP was not significantly associated with PANSS scores (24, 30, 31). Of note, prior studies have shown that the use of antipsychotic medications at the time of data collection does not significantly influence the levels of CRP in schizophrenia (17, 29).

Nonetheless, inflammatory processes appear to play a key role in schizophrenia (3, 5, 10, 15, 17), and CRP has been widely regarded as a state marker in schizophrenia alongside other cytokines like tumor necrosis factor-alpha (1). The current genetic-inflammatory-vascular hypothesis of schizophrenia suggests that a chronic systemic inflammatory state causes CNS-localized microvascular damage leading to a dysregulation of CNS blood-flow homeostasis as well as a breach in the blood–brain barrier (11). Findings from animal models also suggest that elevated levels of markers of inflammation including cytokines could alter neural circuits in the prenatal and postnatal developing brain and predispose to the emergence of psychosis in adulthood. Moreover, systemic pro-inflammatory pathways have been observed to directly alter CNS dopaminergic systems and indirectly modify glutamatergic systems *via* the metabolism of tryptophan and the subsequent increase in CNS-localized kynurenine (secondary to the activity of tryptophan 2,3-dioxygenase and indoleamine 2,3-dioxygenase), which is then converted to kynurenic acid, an NMDA receptor antagonist (1, 5, 41, 42). Importantly, dysregulation of the kynurenine pathway and NMDA dysfunction have been implicated in the pathophysiology of psychosis in schizophrenia and represents an important area of further scientific exploration (5, 41–44). Our findings correlating CRP with psychopathology support and contribute to the hypothesized influence of systemic inflammation upon psychosis in schizophrenia.

Moreover, with regard to prior developmental studies of inflammation and schizophrenia, one would speculate that the current findings are very interesting considering that the negative symptoms often arise early in disease progression, and CRP has been implicated as an early predictor of subsequent development of schizophrenia (18). Some authors have gone so far as to regard CRP as a causative component of schizophrenia (19) alongside other inflammatory molecules such as IL-8 and TNF-alpha, which may alter neurodevelopment (1). Our finding of a correlation between CRP and negative symptoms is also interesting given the overlap of negative symptoms with cognitive symptoms (45), and a relatively consistent finding in several studies is that higher CRP correlates with worse cognitive function in schizophrenia (19, 24, 30, 46). However, our study failed to find an association between CRP and cognition as assessed by the NIH Toolbox Cognitive Test Battery. A possible explanation for the failure to find an association is the relatively small sample size.

The main strength of this study is the adjustment made for potential confounders, an approach lacking in many of the previous studies. The limitations of this study include the cross-sectional design, the relatively small sample size, and the inability to adjust for other potential confounders such as nicotine use or socioeconomic and educational status.

In conclusion, this study investigates a growing area of inquiry within the realm of schizophrenia research, and might be strengthened by increasing sample size, and through the longitudinal study of CRP in patients with schizophrenia over time, ideally from the onset of disease through late progression. If our findings are replicated, and CRP becomes established as a predictor of negative symptom severity, future studies should aim to elucidate the underlying pathophysiological mechanisms of the association, as this could inform the development of novel treatments for negative symptoms of schizophrenia.

## Ethics Statement

The institutional review board of The University of Texas Health Science Center, Houston, Texas approved this study. This research was completed in conformity with the latest version of the Declaration of Helsinki. All study participants completed a written informed consent form after the study was described to them.

## Author Contributions

All individuals meeting authorship criteria are listed as authors, and all certify that they contributed sufficiently to take responsibility for the content. OO contributed to the conception and design of the work, and with data analysis. OO, TB, AT, and RC contributed to the drafting of the manuscript as well as critical revision and final approval of the version to be published.

## Conflict of Interest Statement

This research was conducted in the absence of any commercial or financial relationships that could be regarded as a potential conflict of interest. The authors take complete responsibility for the integrity of the data and the accuracy of the data analysis.
